# 3-{1-[4-(2-Methyl­prop­yl)phen­yl]eth­yl}-1-(morpholinometh­yl)-4-(4-nitro­benzyl­ideneamino)-1*H*-1,2,4-triazole-5(4*H*)-thione

**DOI:** 10.1107/S1600536809003870

**Published:** 2009-02-11

**Authors:** Hoong-Kun Fun, Suchada Chantrapromma, K. V. Sujith, B. Kalluraya

**Affiliations:** a X-ray Crystallography Unit, School of Physics, Universiti Sains Malaysia, 11800 USM, Penang, Malaysia; b Crystal Materials Research Unit, Department of Chemistry, Faculty of Science, Prince of Songkla University, Hat-Yai, Songkhla 90112, Thailand; c Department of Studies in Chemistry, Mangalore University, Mangalagangotri, Mangalore 574 199, India

## Abstract

The title ibuprofen-containing Mannich derivative, C_26_H_32_N_6_O_3_S, crystallizes with two independent mol­ecules in the asymmetric unit. The morpholine ring in each mol­ecule adopts a chair conformation. The 1,2,4-triazole ring forms dihedral angles of 2.13 (10) and 75.52 (10)° with the two substituted benzene rings in one mol­ecule, with corresponding values of 19.36 (11)° and 89.03 (10)° in the other. The nitro groups are twisted from the attached benzene ring in each mol­ecule. In the crystal packing, mol­ecules are linked into supra­molecular chains *via* weak C—H⋯O, C—H⋯N and C—H⋯S inter­actions, and C—H⋯π and N—O⋯π links also occur.

## Related literature

For values of bond lengths, see Allen *et al.* (1987 ▶). For ring conformations, see Cremer & Pople (1975 ▶). For related structures see, for example, Fun *et al.* (2008 ▶). For background to the activities and applications of Mannich derivatives. see, for example, Ferlin *et al.* (2002 ▶); Holla *et al.* (2003 ▶); Joshi *et al.* (2004 ▶); Karthikeyan *et al.* (2006 ▶); Kawail *et al.* (2005 ▶); Klasser & Epstein, (2005 ▶); Lopes *et al.* (2004 ▶); Malinka *et al.* (2005 ▶); Raman *et al.* (2004 ▶); Tramontini *et al.* (1988 ▶).
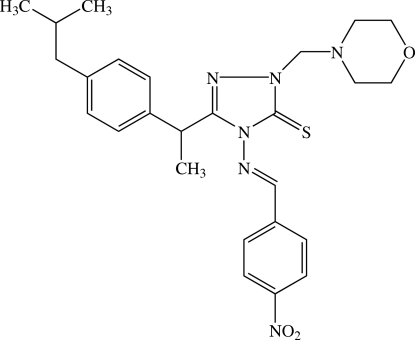


## Experimental

### 

#### Crystal data

C_26_H_32_N_6_O_3_S
*M* *_r_* = 508.65
Triclinic, 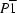

*a* = 10.3104 (1) Å
*b* = 16.9650 (2) Å
*c* = 17.1863 (2) Å
α = 62.656 (1)°
β = 79.907 (1)°
γ = 78.307 (1)°
*V* = 2603.16 (6) Å^3^
*Z* = 4
Mo *K*α radiation
μ = 0.16 mm^−1^
*T* = 100.0 (1) K
0.44 × 0.25 × 0.15 mm

#### Data collection

Bruker APEXII CCD area-detector diffractometer
Absorption correction: multi-scan (*SADABS*; Bruker, 2005 ▶) *T*_min_ = 0.931, *T*_max_ = 0.976
50102 measured reflections
15162 independent reflections
11421 reflections with *I* > 2σ(*I*)
*R*_int_ = 0.044

#### Refinement

*R*[*F* ^2^ > 2σ(*F* ^2^)] = 0.054
*wR*(*F* ^2^) = 0.161
*S* = 1.05
15162 reflections
655 parameters
H-atom parameters constrained
Δρ_max_ = 1.23 e Å^−3^
Δρ_min_ = −0.55 e Å^−3^

### 

Data collection: *APEX2* (Bruker, 2005 ▶); cell refinement: *SAINT* (Bruker, 2005 ▶); data reduction: *SAINT*; program(s) used to solve structure: *SHELXTL* (Sheldrick, 2008 ▶); program(s) used to refine structure: *SHELXTL*; molecular graphics: *SHELXTL*; software used to prepare material for publication: *SHELXTL* and *PLATON* (Spek, 2003 ▶).

## Supplementary Material

Crystal structure: contains datablocks global, I. DOI: 10.1107/S1600536809003870/tk2368sup1.cif

Structure factors: contains datablocks I. DOI: 10.1107/S1600536809003870/tk2368Isup2.hkl

Additional supplementary materials:  crystallographic information; 3D view; checkCIF report

## Figures and Tables

**Table 1:** Hydrogen-bond geometry (Å, °)

*D*—H⋯*A*	*D*—H	H⋯*A*	*D*⋯*A*	*D*—H⋯*A*
C4*A*—H4*AB*⋯O3*A*^i^	0.97	2.53	3.450 (3)	157
C5*A*—H5*AB*⋯O3*B*^ii^	0.97	2.56	3.221 (3)	126
C8*A*—H8*AA*⋯S1*A*	0.93	2.43	3.186 (2)	138
C13*A*—H13*A*⋯O3*A*^iii^	0.93	2.43	3.064 (3)	125
C13*B*—H13*B*⋯N1*A*^iv^	0.93	2.55	3.332 (2)	141
C15*A*—H15*A*⋯O1*A*^v^	0.98	2.27	3.212 (2)	161
C8*B*—H8*BA*⋯S1*B*	0.93	2.55	3.1823 (19)	125
C22*B*—H22*D*⋯S1*A*^iv^	0.97	2.86	3.801 (2)	163
N6*A*—O2*A*⋯*Cg*1^i^	1.23 (1)	3.00 (1)	3.6928 (19)	115 (1)
C11*A*—H11*A*⋯*Cg*2^i^	0.93	2.52	3.405 (2)	158

Symmetry codes: (i) 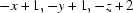; (ii) 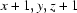; (iii) 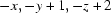; (iv) 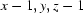; (v) 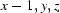.

**Fig. 1.**
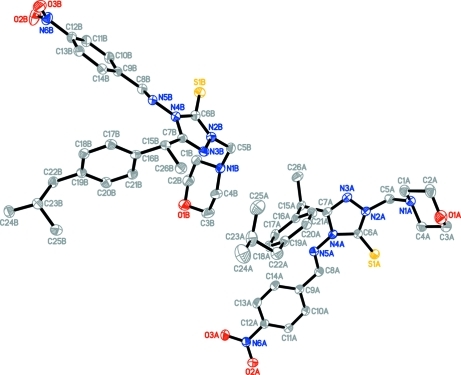
The structure of (I), showing 50% probability displacement ellipsoids and the atom-numbering scheme. Hydrogen atoms are omitted for clarify.

**Fig. 2.**
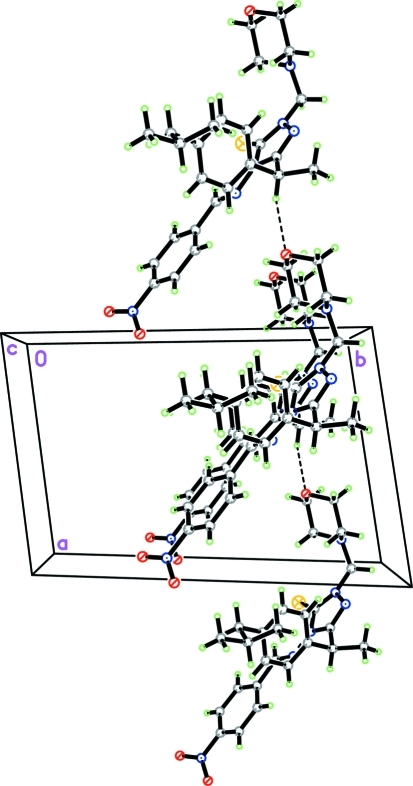
A packing diagram for (I), viewed along the c axis, showing supramolecular chains along the a axis. Weak C—H···O interactions are shown as dashed lines.

## supplementary crystallographic information

### Comment

Non-steroidal anti-inflammatory drugs (NSAIDs) such as ibuprofen are widely used in the treatment of pain and inflammation (Kawail *et al.*, 2005; Klasser & Epstein, 2005). The Mannich reaction is a three-component condensation reaction involving active hydrogen-containing compounds, formaldehyde and a secondary amine (Raman *et al.*, 2004). Mannich bases have been reported as potential biological agents. Thus, they find applications as antitubercular (Joshi *et al.*, 2004), antimalarial (Lopes *et al.*, 2004), vasorelaxing (Ferlin *et al.*, 2002), anticancer (Holla *et al.*, 2003), and analgesic drugs (Malinka *et al.*, 2005). They are also used in the polymer industry as paints and surface active agents (Tramontini *et al.*, 1988). Some Mannich bases are reported to exhibit activity *in vitro* against murine P388 lymphocytic leukemia cells (Karthikeyan *et al.*, 2006). According to our previous work (Fun *et al.*, 2008), we are interested in the synthesis, biological activities and crystal structures of ibuprofen- containing Mannich derivatives. Prompted by the biological activities of these derivatives, we herein report the synthesis and the crystal structure determination of the title compound, (I).

In the asymmetric unit of (I), Fig. 1, there are two independent molecules, designated *A* and *B*. The morpholine ring (C1–C4//N1/O1) in each molecule is in the standard chair conformation (Cremer & Pople, 1975), and is (-)-*anti*-clinal with respect to the 1,2,4-triazole ring as seen in the torsion angle C6–N2–C5–N1 = -98.3 (2)° in molecule *A*; -90.8 (2)° in molecule *B*. The 1,2,4-triazole ring forms dihedral angles of 2.13 (10)° and 75.52 (10)° with the C9–C14 and C16–C21 benzene rings, respectively in molecule *A*, with corresponding values of 19.36 (11)° and 89.03 (10)° for molecule *B*. These data indicate the 4-nitrophenyl unit is essentially co-planar with the 1,2,4-triazole ring in molecule *A* whereas it is twisted in molecule *B*. In the 4-nitrophenyl unit of each molecule, the nitro group is twisted from the mean plane of the C9–C14 ring as shown by the dihedral angle formed between the mean planes through C12/N6/O2/O3 and C19–C14 ring of 21.63 (11)° in molecule *A*; the comparable angle is 27.96 (13)° for molecule *B*. The isobutyl substituent (C22–C25) in each molecule is (-)-*syn*-clinal with respect to the C16–C21 ring with the torsion angle C18–C19–C22–C23 = -75.7 (2)° in molecule *A* compared with -74.2 (2)° in molecule *B*. Bond lengths and angles in molecules *A* and *B* are slightly different but all are in normal ranges (Allen *et al.*, 1987) and comparable to those in a related structure (Fun *et al.*, 2008).

In the crystal packing, weak C—H···O interactions (Table 1, Fig. 2) link the molecules into chains along the *a*-axis. The crystal is further stabilized by weak C—H···O, C—H···π and N—O···π interactions (Table 1); *Cg*_1_ and *Cg*_2_ are the centroids of N2A–N3A–C7A–N4A–C6A and C16A–C21A rings, respectively.

### Experimental

Compound (I) was obtained by the aminomethylation of its corresponding Schiff base, which was obtained by refluxing (3 h) 4-amino-5-[1-(4-isobutylphenyl)ethyl]-4*H*-1,2,4-triazole-3-thiol (0.01 mol) and 4-nitro benzaldehyde (0.01 mol) in ethanol (50 ml) with the addition of 3 drops of concentrated sulfuric acid. A mixture of the resulting Schiff base (0.01 mol), formaldehyde (40%, 1 ml) and morpholine (0.01 mol) in ethanol (50 ml) was stirred at room temperature for 20 h. The solid product obtained was collected by filtration, washed with ethanol and dried. Yellow block-shaped single crystals were obtained from an ethanol/*N*,*N*-dimethylformamide (DMF) (2:1) solution by slow evaporation (Yield 85%, *M*.p. 387 K).

### Refinement

All H atoms were placed in their calculated positions with d(C—H) = 0.93-0.98 Å, and with *U*_iso_ = 1.2-1.5*U*_eq_(C). The rotating group model was used for the methyl groups. The highest residual electron density peak was located 0.86 Å from S1B and the deepest hole was located 0.54 Å from S1B.

### Crystal data

**Table:**

C_26_H_32_N_6_O_3_S	*Z* = 4
*M**_r_* = 508.65	*F*(000) = 1080
Triclinic, *P*1	*D*_x_ = 1.298 Mg m^−^^3^
Hall symbol: -P 1	Melting point: 387 K
*a* = 10.3104 (1) Å	Mo *K*α radiation, λ = 0.71073 Å
*b* = 16.9650 (2) Å	Cell parameters from 15162 reflections
*c* = 17.1863 (2) Å	θ = 1.3–30.0°
α = 62.656 (1)°	µ = 0.16 mm^−^^1^
β = 79.907 (1)°	*T* = 100 K
γ = 78.307 (1)°	Block, yellow
*V* = 2603.16 (6) Å^3^	0.44 × 0.25 × 0.15 mm

### Data collection

**Table:**

Bruker APEXII CCD area-detector diffractometer	15162 independent reflections
Radiation source: fine-focus sealed tube	11421 reflections with *I* > 2σ(*I*)
graphite	*R*_int_ = 0.044
Detector resolution: 8.33 pixels mm^-1^	θ_max_ = 30.0°, θ_min_ = 1.3°
ω scans	*h* = −14→14
Absorption correction: multi-scan (*SADABS*; Bruker, 2005)	*k* = −23→23
*T*_min_ = 0.931, *T*_max_ = 0.976	*l* = −24→24
50102 measured reflections	

### Refinement

**Table:**

Refinement on *F*^2^	Primary atom site location: structure-invariant direct methods
Least-squares matrix: full	Secondary atom site location: difference Fourier map
*R*[*F*^2^ > 2σ(*F*^2^)] = 0.054	Hydrogen site location: inferred from neighbouring sites
*wR*(*F*^2^) = 0.161	H-atom parameters constrained
*S* = 1.05	*w* = 1/[σ^2^(*F*_o_^2^) + (0.079*P*)^2^ + 1.217*P*] where *P* = (*F*_o_^2^ + 2*F*_c_^2^)/3
15162 reflections	(Δ/σ)_max_ = 0.001
655 parameters	Δρ_max_ = 1.23 e Å^−^^3^
0 restraints	Δρ_min_ = −0.55 e Å^−^^3^

### Special details

**Table:**

Experimental. The low-temperature data was collected with the Oxford Cyrosystem Cobra low-temperature attachment.
Geometry. All e.s.d.'s (except the e.s.d. in the dihedral angle between two l.s. planes) are estimated using the full covariance matrix. The cell e.s.d.'s are taken into account individually in the estimation of e.s.d.'s in distances, angles and torsion angles; correlations between e.s.d.'s in cell parameters are only used when they are defined by crystal symmetry. An approximate (isotropic) treatment of cell e.s.d.'s is used for estimating e.s.d.'s involving l.s. planes.
Refinement. Refinement of *F*^2^ against ALL reflections. The weighted *R*-factor *wR* and goodness of fit *S* are based on *F*^2^, conventional *R*-factors *R* are based on *F*, with *F* set to zero for negative *F*^2^. The threshold expression of *F*^2^ > σ(*F*^2^) is used only for calculating *R*-factors(gt) *etc*. and is not relevant to the choice of reflections for refinement. *R*-factors based on *F*^2^ are statistically about twice as large as those based on *F*, and *R*- factors based on ALL data will be even larger.

### Fractional atomic coordinates and isotropic or equivalent isotropic displacement parameters (Å^2^)

**Table:**

	*x*	*y*	*z*	*U*_iso_*/*U*_eq_	
S1A	0.78547 (5)	0.25240 (3)	1.21421 (3)	0.02654 (11)	
O1A	1.29830 (15)	0.18812 (11)	1.04587 (11)	0.0379 (4)	
O2A	0.08384 (14)	0.68658 (9)	1.03927 (10)	0.0290 (3)	
O3A	−0.01242 (15)	0.60984 (10)	1.00038 (11)	0.0378 (4)	
N1A	1.08560 (15)	0.09773 (11)	1.15711 (11)	0.0242 (3)	
N2A	0.84566 (15)	0.14004 (10)	1.13677 (10)	0.0208 (3)	
N3A	0.80185 (15)	0.11781 (10)	1.07871 (10)	0.0215 (3)	
N4A	0.67012 (14)	0.23297 (10)	1.09020 (10)	0.0195 (3)	
N5A	0.55767 (15)	0.29644 (10)	1.07082 (10)	0.0211 (3)	
N6A	0.08197 (15)	0.62115 (11)	1.02708 (10)	0.0243 (3)	
C1A	1.12567 (19)	0.09867 (15)	1.07070 (14)	0.0300 (4)	
H1AA	1.0737	0.1484	1.0268	0.036*	
H1AB	1.1101	0.0435	1.0722	0.036*	
C2A	1.2725 (2)	0.10822 (17)	1.04711 (16)	0.0355 (5)	
H2AA	1.3240	0.0569	1.0898	0.043*	
H2AB	1.3001	0.1094	0.9897	0.043*	
C3A	1.2609 (2)	0.18562 (15)	1.13062 (15)	0.0311 (4)	
H3AA	1.2803	0.2396	1.1299	0.037*	
H3AB	1.3129	0.1347	1.1734	0.037*	
C4A	1.11489 (19)	0.17850 (13)	1.15776 (13)	0.0257 (4)	
H4AA	1.0923	0.1753	1.2163	0.031*	
H4AB	1.0621	0.2311	1.1173	0.031*	
C5A	0.95186 (18)	0.07875 (12)	1.19086 (12)	0.0241 (4)	
H5AA	0.9341	0.0787	1.2482	0.029*	
H5AB	0.9472	0.0187	1.1995	0.029*	
C6A	0.76712 (17)	0.20949 (12)	1.14700 (11)	0.0200 (3)	
C7A	0.69639 (17)	0.17506 (12)	1.05098 (11)	0.0200 (3)	
C8A	0.53313 (18)	0.35138 (12)	1.10518 (12)	0.0226 (3)	
H8AA	0.5901	0.3483	1.1432	0.027*	
C9A	0.41469 (17)	0.41923 (12)	1.08419 (11)	0.0206 (3)	
C10A	0.39129 (18)	0.47859 (12)	1.12251 (12)	0.0225 (3)	
H10A	0.4492	0.4728	1.1612	0.027*	
C11A	0.28338 (17)	0.54563 (12)	1.10365 (12)	0.0216 (3)	
H11A	0.2685	0.5857	1.1283	0.026*	
C12A	0.19824 (17)	0.55113 (12)	1.04683 (12)	0.0217 (3)	
C13A	0.21717 (19)	0.49364 (13)	1.00776 (13)	0.0255 (4)	
H13A	0.1577	0.4992	0.9700	0.031*	
C14A	0.32678 (18)	0.42769 (13)	1.02631 (12)	0.0241 (4)	
H14A	0.3421	0.3888	1.0002	0.029*	
C15A	0.61192 (17)	0.17984 (12)	0.98601 (12)	0.0215 (3)	
H15A	0.5188	0.1954	1.0046	0.026*	
C16A	0.64620 (17)	0.25239 (12)	0.89457 (12)	0.0209 (3)	
C17A	0.54930 (17)	0.32166 (14)	0.84976 (13)	0.0247 (4)	
H17A	0.4632	0.3249	0.8768	0.030*	
C18A	0.57976 (18)	0.38588 (13)	0.76534 (13)	0.0250 (4)	
H18A	0.5136	0.4318	0.7366	0.030*	
C19A	0.70763 (18)	0.38326 (13)	0.72233 (12)	0.0234 (4)	
C20A	0.80489 (18)	0.31444 (13)	0.76766 (12)	0.0241 (4)	
H20A	0.8911	0.3117	0.7408	0.029*	
C21A	0.77525 (17)	0.24982 (13)	0.85243 (12)	0.0225 (3)	
H21A	0.8417	0.2044	0.8815	0.027*	
C22A	0.7373 (2)	0.45101 (14)	0.62909 (13)	0.0306 (4)	
H22A	0.8328	0.4506	0.6163	0.037*	
H22B	0.6977	0.5102	0.6232	0.037*	
C23A	0.6842 (2)	0.43256 (16)	0.56158 (14)	0.0347 (5)	
H23A	0.5879	0.4328	0.5757	0.042*	
C24A	0.7086 (3)	0.5073 (2)	0.46935 (16)	0.0527 (7)	
H24A	0.6723	0.4964	0.4279	0.079*	
H24B	0.8026	0.5086	0.4541	0.079*	
H24C	0.6663	0.5637	0.4679	0.079*	
C25A	0.7450 (3)	0.34170 (18)	0.56554 (16)	0.0419 (5)	
H25A	0.7098	0.3327	0.5226	0.063*	
H25B	0.7238	0.2956	0.6232	0.063*	
H25C	0.8398	0.3393	0.5534	0.063*	
C26A	0.6264 (2)	0.08819 (14)	0.98539 (14)	0.0299 (4)	
H26A	0.5989	0.0444	1.0429	0.045*	
H26B	0.7177	0.0706	0.9693	0.045*	
H26C	0.5717	0.0923	0.9435	0.045*	
S1B	0.41995 (5)	−0.06478 (3)	0.71988 (3)	0.02460 (10)	
O1B	0.15427 (15)	0.26352 (11)	0.76303 (12)	0.0394 (4)	
O2B	−0.0664 (2)	−0.15572 (14)	0.39243 (12)	0.0545 (5)	
O3B	0.09808 (18)	−0.12340 (11)	0.28982 (11)	0.0414 (4)	
N1B	0.39755 (15)	0.14654 (11)	0.76939 (11)	0.0239 (3)	
N2B	0.49615 (14)	0.10079 (10)	0.65296 (10)	0.0204 (3)	
N3B	0.51644 (15)	0.17613 (10)	0.57463 (10)	0.0212 (3)	
N4B	0.42374 (14)	0.08104 (10)	0.55504 (9)	0.0185 (3)	
N5B	0.36838 (14)	0.05378 (10)	0.50452 (10)	0.0194 (3)	
N6B	0.0391 (2)	−0.12622 (12)	0.35983 (12)	0.0340 (4)	
C1B	0.26918 (18)	0.12013 (14)	0.77168 (13)	0.0262 (4)	
H1BA	0.2721	0.0559	0.8069	0.031*	
H1BB	0.2526	0.1343	0.7125	0.031*	
C2B	0.1578 (2)	0.16859 (16)	0.81025 (16)	0.0349 (5)	
H2BA	0.0735	0.1527	0.8085	0.042*	
H2BB	0.1699	0.1497	0.8713	0.042*	
C3B	0.2782 (2)	0.28701 (16)	0.76481 (17)	0.0366 (5)	
H3BA	0.2941	0.2676	0.8253	0.044*	
H3BB	0.2746	0.3517	0.7344	0.044*	
C4B	0.3912 (2)	0.24449 (13)	0.72165 (14)	0.0290 (4)	
H4BA	0.3769	0.2645	0.6607	0.035*	
H4BB	0.4744	0.2620	0.7229	0.035*	
C5B	0.50905 (18)	0.09861 (13)	0.73837 (12)	0.0236 (4)	
H5BA	0.5209	0.0366	0.7822	0.028*	
H5BB	0.5886	0.1237	0.7329	0.028*	
C6B	0.44259 (16)	0.03925 (12)	0.64404 (12)	0.0200 (3)	
C7B	0.47454 (16)	0.16148 (12)	0.51579 (12)	0.0185 (3)	
C8B	0.28102 (17)	0.00087 (12)	0.54485 (12)	0.0210 (3)	
H8BA	0.2578	−0.0181	0.6049	0.025*	
C9B	0.21784 (16)	−0.02954 (12)	0.49513 (12)	0.0196 (3)	
C10B	0.13500 (18)	−0.09569 (13)	0.54181 (13)	0.0243 (4)	
H10B	0.1209	−0.1190	0.6028	0.029*	
C11B	0.07368 (19)	−0.12675 (13)	0.49744 (13)	0.0267 (4)	
H11B	0.0164	−0.1695	0.5276	0.032*	
C12B	0.10036 (19)	−0.09221 (13)	0.40720 (13)	0.0252 (4)	
C13B	0.18152 (18)	−0.02639 (12)	0.35903 (12)	0.0234 (3)	
H13B	0.1974	−0.0047	0.2982	0.028*	
C14B	0.23843 (17)	0.00630 (12)	0.40353 (12)	0.0216 (3)	
H14B	0.2904	0.0522	0.3724	0.026*	
C15B	0.47645 (17)	0.22221 (12)	0.41892 (12)	0.0199 (3)	
H15B	0.5191	0.1867	0.3874	0.024*	
C16B	0.33609 (17)	0.25916 (11)	0.39207 (11)	0.0190 (3)	
C17B	0.29910 (19)	0.26082 (12)	0.31672 (12)	0.0235 (4)	
H17B	0.3607	0.2379	0.2832	0.028*	
C18B	0.17122 (19)	0.29640 (12)	0.29164 (12)	0.0238 (4)	
H18B	0.1487	0.2976	0.2410	0.029*	
C19B	0.07559 (17)	0.33051 (12)	0.34104 (12)	0.0206 (3)	
C20B	0.11367 (18)	0.32937 (13)	0.41542 (12)	0.0236 (4)	
H20B	0.0521	0.3523	0.4490	0.028*	
C21B	0.24170 (18)	0.29470 (13)	0.44049 (12)	0.0230 (3)	
H21B	0.2648	0.2952	0.4902	0.028*	
C22B	−0.06331 (17)	0.36802 (12)	0.31357 (12)	0.0226 (3)	
H22C	−0.1209	0.3726	0.3628	0.027*	
H22D	−0.0955	0.3263	0.2999	0.027*	
C23B	−0.07409 (18)	0.46057 (12)	0.23353 (12)	0.0222 (3)	
H23B	−0.0204	0.4546	0.1830	0.027*	
C24B	−0.21841 (19)	0.49194 (14)	0.21189 (14)	0.0283 (4)	
H24D	−0.2249	0.5496	0.1619	0.042*	
H24E	−0.2731	0.4962	0.2615	0.042*	
H24F	−0.2481	0.4497	0.1986	0.042*	
C25B	−0.02021 (19)	0.52833 (13)	0.25018 (13)	0.0261 (4)	
H25D	−0.0315	0.5860	0.2005	0.039*	
H25E	0.0726	0.5094	0.2586	0.039*	
H25F	−0.0678	0.5321	0.3019	0.039*	
C26B	0.55916 (19)	0.29752 (13)	0.39370 (13)	0.0259 (4)	
H26D	0.6477	0.2720	0.4106	0.039*	
H26E	0.5187	0.3337	0.4235	0.039*	
H26F	0.5628	0.3341	0.3313	0.039*	

### Atomic displacement parameters (Å^2^)

**Table:**

	*U*^11^	*U*^22^	*U*^33^	*U*^12^	*U*^13^	*U*^23^
S1A	0.0317 (2)	0.0282 (2)	0.0251 (2)	−0.00099 (19)	−0.00806 (19)	−0.0156 (2)
O1A	0.0279 (7)	0.0444 (9)	0.0403 (9)	−0.0088 (7)	0.0044 (7)	−0.0188 (8)
O2A	0.0319 (7)	0.0213 (6)	0.0328 (8)	−0.0038 (6)	−0.0056 (6)	−0.0101 (6)
O3A	0.0293 (7)	0.0294 (8)	0.0534 (10)	−0.0063 (6)	−0.0183 (7)	−0.0113 (7)
N1A	0.0240 (7)	0.0240 (8)	0.0242 (8)	−0.0025 (6)	−0.0026 (6)	−0.0104 (7)
N2A	0.0237 (7)	0.0205 (7)	0.0192 (7)	−0.0059 (6)	−0.0021 (6)	−0.0085 (6)
N3A	0.0242 (7)	0.0228 (7)	0.0204 (7)	−0.0082 (6)	0.0006 (6)	−0.0108 (6)
N4A	0.0207 (7)	0.0215 (7)	0.0179 (7)	−0.0060 (6)	0.0000 (5)	−0.0096 (6)
N5A	0.0204 (7)	0.0215 (7)	0.0207 (7)	−0.0044 (6)	0.0000 (6)	−0.0089 (6)
N6A	0.0233 (7)	0.0216 (7)	0.0225 (8)	−0.0067 (6)	−0.0038 (6)	−0.0031 (6)
C1A	0.0236 (9)	0.0387 (11)	0.0324 (11)	−0.0002 (8)	−0.0019 (8)	−0.0214 (9)
C2A	0.0245 (9)	0.0480 (13)	0.0381 (12)	0.0005 (9)	−0.0016 (8)	−0.0252 (11)
C3A	0.0263 (9)	0.0316 (10)	0.0369 (11)	−0.0060 (8)	−0.0009 (8)	−0.0162 (9)
C4A	0.0260 (9)	0.0228 (9)	0.0274 (10)	−0.0034 (7)	−0.0023 (7)	−0.0103 (8)
C5A	0.0261 (9)	0.0208 (8)	0.0221 (9)	−0.0023 (7)	−0.0035 (7)	−0.0067 (7)
C6A	0.0222 (8)	0.0194 (8)	0.0170 (8)	−0.0052 (7)	−0.0015 (6)	−0.0059 (7)
C7A	0.0211 (8)	0.0222 (8)	0.0185 (8)	−0.0086 (7)	0.0036 (6)	−0.0101 (7)
C8A	0.0242 (8)	0.0251 (9)	0.0199 (8)	−0.0063 (7)	−0.0010 (7)	−0.0102 (7)
C9A	0.0219 (8)	0.0225 (8)	0.0181 (8)	−0.0078 (7)	0.0013 (6)	−0.0085 (7)
C10A	0.0235 (8)	0.0260 (9)	0.0213 (9)	−0.0050 (7)	−0.0042 (7)	−0.0118 (7)
C11A	0.0229 (8)	0.0223 (8)	0.0212 (8)	−0.0063 (7)	−0.0006 (7)	−0.0102 (7)
C12A	0.0210 (8)	0.0211 (8)	0.0211 (8)	−0.0077 (7)	−0.0006 (7)	−0.0063 (7)
C13A	0.0276 (9)	0.0281 (9)	0.0236 (9)	−0.0084 (8)	−0.0056 (7)	−0.0108 (8)
C14A	0.0278 (9)	0.0271 (9)	0.0217 (9)	−0.0081 (7)	−0.0019 (7)	−0.0127 (8)
C15A	0.0188 (7)	0.0277 (9)	0.0237 (9)	−0.0064 (7)	0.0001 (7)	−0.0156 (8)
C16A	0.0202 (8)	0.0257 (9)	0.0232 (9)	−0.0044 (7)	−0.0017 (7)	−0.0158 (7)
C17A	0.0161 (7)	0.0379 (10)	0.0291 (10)	−0.0031 (7)	−0.0017 (7)	−0.0229 (9)
C18A	0.0223 (8)	0.0303 (9)	0.0271 (9)	0.0031 (7)	−0.0087 (7)	−0.0169 (8)
C19A	0.0253 (8)	0.0270 (9)	0.0219 (9)	−0.0046 (7)	−0.0033 (7)	−0.0135 (8)
C20A	0.0200 (8)	0.0302 (9)	0.0249 (9)	−0.0032 (7)	0.0005 (7)	−0.0156 (8)
C21A	0.0194 (8)	0.0235 (8)	0.0253 (9)	0.0006 (7)	−0.0024 (7)	−0.0128 (7)
C22A	0.0358 (10)	0.0306 (10)	0.0243 (10)	−0.0064 (9)	−0.0033 (8)	−0.0103 (8)
C23A	0.0316 (10)	0.0495 (13)	0.0220 (10)	−0.0044 (10)	−0.0032 (8)	−0.0151 (9)
C24A	0.0591 (16)	0.0597 (17)	0.0254 (12)	−0.0043 (14)	−0.0077 (11)	−0.0072 (11)
C25A	0.0485 (13)	0.0537 (15)	0.0329 (12)	−0.0124 (12)	0.0026 (10)	−0.0271 (11)
C26A	0.0302 (9)	0.0316 (10)	0.0365 (11)	−0.0084 (8)	0.0002 (8)	−0.0218 (9)
S1B	0.0269 (2)	0.0210 (2)	0.0219 (2)	−0.00602 (17)	0.00018 (17)	−0.00583 (18)
O1B	0.0294 (7)	0.0398 (9)	0.0595 (11)	0.0027 (7)	−0.0061 (7)	−0.0330 (8)
O2B	0.0632 (12)	0.0635 (12)	0.0423 (10)	−0.0376 (10)	−0.0172 (9)	−0.0123 (9)
O3B	0.0617 (11)	0.0308 (8)	0.0368 (9)	−0.0003 (8)	−0.0182 (8)	−0.0170 (7)
N1B	0.0223 (7)	0.0268 (8)	0.0259 (8)	−0.0030 (6)	−0.0033 (6)	−0.0143 (7)
N2B	0.0200 (7)	0.0225 (7)	0.0187 (7)	−0.0027 (6)	−0.0006 (6)	−0.0097 (6)
N3B	0.0208 (7)	0.0199 (7)	0.0230 (8)	−0.0015 (6)	−0.0017 (6)	−0.0102 (6)
N4B	0.0186 (6)	0.0204 (7)	0.0169 (7)	−0.0041 (6)	−0.0004 (5)	−0.0084 (6)
N5B	0.0189 (6)	0.0196 (7)	0.0206 (7)	−0.0023 (6)	−0.0041 (6)	−0.0090 (6)
N6B	0.0496 (11)	0.0242 (8)	0.0289 (9)	−0.0095 (8)	−0.0165 (8)	−0.0060 (7)
C1B	0.0246 (9)	0.0281 (9)	0.0293 (10)	−0.0051 (8)	−0.0015 (7)	−0.0153 (8)
C2B	0.0279 (10)	0.0427 (12)	0.0428 (13)	−0.0046 (9)	0.0005 (9)	−0.0278 (11)
C3B	0.0344 (11)	0.0352 (11)	0.0511 (14)	−0.0029 (9)	−0.0021 (10)	−0.0296 (11)
C4B	0.0318 (10)	0.0255 (9)	0.0338 (11)	−0.0052 (8)	−0.0006 (8)	−0.0170 (8)
C5B	0.0228 (8)	0.0274 (9)	0.0228 (9)	−0.0019 (7)	−0.0051 (7)	−0.0125 (8)
C6B	0.0168 (7)	0.0232 (8)	0.0194 (8)	−0.0033 (6)	0.0013 (6)	−0.0097 (7)
C7B	0.0157 (7)	0.0190 (8)	0.0216 (8)	−0.0017 (6)	0.0005 (6)	−0.0106 (7)
C8B	0.0201 (8)	0.0239 (8)	0.0195 (8)	−0.0032 (7)	−0.0005 (6)	−0.0102 (7)
C9B	0.0173 (7)	0.0190 (8)	0.0219 (8)	−0.0016 (6)	−0.0030 (6)	−0.0086 (7)
C10B	0.0243 (8)	0.0241 (9)	0.0205 (9)	−0.0064 (7)	−0.0035 (7)	−0.0047 (7)
C11B	0.0289 (9)	0.0215 (9)	0.0265 (10)	−0.0083 (7)	−0.0076 (8)	−0.0042 (7)
C12B	0.0280 (9)	0.0224 (9)	0.0259 (9)	−0.0040 (7)	−0.0087 (7)	−0.0089 (7)
C13B	0.0260 (8)	0.0226 (8)	0.0199 (8)	−0.0029 (7)	−0.0030 (7)	−0.0079 (7)
C14B	0.0191 (8)	0.0207 (8)	0.0223 (9)	−0.0037 (7)	−0.0011 (7)	−0.0071 (7)
C15B	0.0205 (8)	0.0181 (8)	0.0206 (8)	−0.0029 (6)	0.0006 (6)	−0.0090 (7)
C16B	0.0219 (8)	0.0155 (7)	0.0192 (8)	−0.0046 (6)	0.0007 (6)	−0.0074 (6)
C17B	0.0281 (9)	0.0229 (8)	0.0201 (9)	−0.0012 (7)	−0.0001 (7)	−0.0118 (7)
C18B	0.0296 (9)	0.0233 (9)	0.0209 (9)	−0.0024 (7)	−0.0052 (7)	−0.0112 (7)
C19B	0.0220 (8)	0.0169 (8)	0.0210 (8)	−0.0054 (6)	−0.0014 (7)	−0.0058 (7)
C20B	0.0229 (8)	0.0258 (9)	0.0226 (9)	−0.0019 (7)	0.0006 (7)	−0.0124 (7)
C21B	0.0240 (8)	0.0264 (9)	0.0214 (9)	−0.0022 (7)	−0.0013 (7)	−0.0138 (7)
C22B	0.0213 (8)	0.0240 (8)	0.0225 (9)	−0.0070 (7)	−0.0022 (7)	−0.0086 (7)
C23B	0.0224 (8)	0.0233 (8)	0.0195 (8)	−0.0014 (7)	−0.0008 (7)	−0.0092 (7)
C24B	0.0260 (9)	0.0302 (10)	0.0289 (10)	0.0015 (8)	−0.0071 (8)	−0.0137 (8)
C25B	0.0250 (9)	0.0228 (9)	0.0286 (10)	−0.0035 (7)	−0.0003 (7)	−0.0105 (8)
C26B	0.0237 (8)	0.0232 (9)	0.0289 (10)	−0.0068 (7)	−0.0003 (7)	−0.0091 (8)

### Geometric parameters (Å, °)

**Table:**

S1A—C6A	1.6747 (17)	S1B—C6B	1.6777 (19)
O1A—C3A	1.423 (3)	O1B—C3B	1.422 (3)
O1A—C2A	1.424 (3)	O1B—C2B	1.428 (3)
O2A—N6A	1.225 (2)	O2B—N6B	1.224 (3)
O3A—N6A	1.228 (2)	O3B—N6B	1.234 (2)
N1A—C5A	1.439 (2)	N1B—C5B	1.439 (2)
N1A—C1A	1.464 (2)	N1B—C1B	1.470 (2)
N1A—C4A	1.467 (2)	N1B—C4B	1.470 (3)
N2A—C6A	1.352 (2)	N2B—C6B	1.352 (2)
N2A—N3A	1.3836 (19)	N2B—N3B	1.385 (2)
N2A—C5A	1.467 (2)	N2B—C5B	1.480 (2)
N3A—C7A	1.297 (2)	N3B—C7B	1.303 (2)
N4A—N5A	1.384 (2)	N4B—C7B	1.383 (2)
N4A—C7A	1.389 (2)	N4B—C6B	1.390 (2)
N4A—C6A	1.391 (2)	N4B—N5B	1.3939 (18)
N5A—C8A	1.280 (2)	N5B—C8B	1.279 (2)
N6A—C12A	1.469 (2)	N6B—C12B	1.467 (2)
C1A—C2A	1.519 (3)	C1B—C2B	1.512 (3)
C1A—H1AA	0.9700	C1B—H1BA	0.9700
C1A—H1AB	0.9700	C1B—H1BB	0.9700
C2A—H2AA	0.9700	C2B—H2BA	0.9700
C2A—H2AB	0.9700	C2B—H2BB	0.9700
C3A—C4A	1.508 (3)	C3B—C4B	1.509 (3)
C3A—H3AA	0.9700	C3B—H3BA	0.9700
C3A—H3AB	0.9700	C3B—H3BB	0.9700
C4A—H4AA	0.9700	C4B—H4BA	0.9700
C4A—H4AB	0.9700	C4B—H4BB	0.9700
C5A—H5AA	0.9700	C5B—H5BA	0.9700
C5A—H5AB	0.9700	C5B—H5BB	0.9700
C7A—C15A	1.497 (2)	C7B—C15B	1.502 (2)
C8A—C9A	1.466 (3)	C8B—C9B	1.469 (2)
C8A—H8AA	0.9300	C8B—H8BA	0.9300
C9A—C10A	1.401 (2)	C9B—C14B	1.396 (2)
C9A—C14A	1.401 (2)	C9B—C10B	1.398 (2)
C10A—C11A	1.383 (3)	C10B—C11B	1.390 (2)
C10A—H10A	0.9300	C10B—H10B	0.9300
C11A—C12A	1.384 (2)	C11B—C12B	1.380 (3)
C11A—H11A	0.9300	C11B—H11B	0.9300
C12A—C13A	1.385 (2)	C12B—C13B	1.385 (3)
C13A—C14A	1.384 (3)	C13B—C14B	1.384 (2)
C13A—H13A	0.9300	C13B—H13B	0.9300
C14A—H14A	0.9300	C14B—H14B	0.9300
C15A—C16A	1.521 (3)	C15B—C16B	1.517 (2)
C15A—C26A	1.536 (2)	C15B—C26B	1.533 (2)
C15A—H15A	0.9800	C15B—H15B	0.9800
C16A—C17A	1.391 (3)	C16B—C21B	1.394 (2)
C16A—C21A	1.402 (2)	C16B—C17B	1.400 (2)
C17A—C18A	1.385 (3)	C17B—C18B	1.388 (3)
C17A—H17A	0.9300	C17B—H17B	0.9300
C18A—C19A	1.397 (3)	C18B—C19B	1.398 (2)
C18A—H18A	0.9300	C18B—H18B	0.9300
C19A—C20A	1.393 (3)	C19B—C20B	1.393 (2)
C19A—C22A	1.504 (3)	C19B—C22B	1.507 (2)
C20A—C21A	1.389 (3)	C20B—C21B	1.388 (3)
C20A—H20A	0.9300	C20B—H20B	0.9300
C21A—H21A	0.9300	C21B—H21B	0.9300
C22A—C23A	1.539 (3)	C22B—C23B	1.539 (3)
C22A—H22A	0.9700	C22B—H22C	0.9700
C22A—H22B	0.9700	C22B—H22D	0.9700
C23A—C25A	1.517 (3)	C23B—C24B	1.525 (3)
C23A—C24A	1.525 (3)	C23B—C25B	1.526 (2)
C23A—H23A	0.9800	C23B—H23B	0.9800
C24A—H24A	0.9600	C24B—H24D	0.9600
C24A—H24B	0.9600	C24B—H24E	0.9600
C24A—H24C	0.9600	C24B—H24F	0.9600
C25A—H25A	0.9600	C25B—H25D	0.9600
C25A—H25B	0.9600	C25B—H25E	0.9600
C25A—H25C	0.9600	C25B—H25F	0.9600
C26A—H26A	0.9600	C26B—H26D	0.9600
C26A—H26B	0.9600	C26B—H26E	0.9600
C26A—H26C	0.9600	C26B—H26F	0.9600
			
C3A—O1A—C2A	109.53 (17)	C3B—O1B—C2B	109.47 (17)
C5A—N1A—C1A	113.59 (15)	C5B—N1B—C1B	113.34 (14)
C5A—N1A—C4A	114.12 (14)	C5B—N1B—C4B	113.71 (15)
C1A—N1A—C4A	110.92 (16)	C1B—N1B—C4B	110.06 (15)
C6A—N2A—N3A	113.39 (14)	C6B—N2B—N3B	113.63 (14)
C6A—N2A—C5A	127.05 (14)	C6B—N2B—C5B	124.40 (15)
N3A—N2A—C5A	118.50 (14)	N3B—N2B—C5B	120.99 (14)
C7A—N3A—N2A	104.86 (14)	C7B—N3B—N2B	104.62 (14)
N5A—N4A—C7A	118.58 (14)	C7B—N4B—C6B	108.92 (14)
N5A—N4A—C6A	133.08 (14)	C7B—N4B—N5B	120.03 (14)
C7A—N4A—C6A	108.27 (14)	C6B—N4B—N5B	131.03 (14)
C8A—N5A—N4A	119.42 (15)	C8B—N5B—N4B	116.47 (14)
O2A—N6A—O3A	123.82 (16)	O2B—N6B—O3B	124.42 (18)
O2A—N6A—C12A	118.39 (15)	O2B—N6B—C12B	117.97 (18)
O3A—N6A—C12A	117.78 (15)	O3B—N6B—C12B	117.60 (18)
N1A—C1A—C2A	108.67 (16)	N1B—C1B—C2B	110.51 (15)
N1A—C1A—H1AA	110.0	N1B—C1B—H1BA	109.5
C2A—C1A—H1AA	110.0	C2B—C1B—H1BA	109.5
N1A—C1A—H1AB	110.0	N1B—C1B—H1BB	109.5
C2A—C1A—H1AB	110.0	C2B—C1B—H1BB	109.5
H1AA—C1A—H1AB	108.3	H1BA—C1B—H1BB	108.1
O1A—C2A—C1A	111.07 (17)	O1B—C2B—C1B	111.46 (18)
O1A—C2A—H2AA	109.4	O1B—C2B—H2BA	109.3
C1A—C2A—H2AA	109.4	C1B—C2B—H2BA	109.3
O1A—C2A—H2AB	109.4	O1B—C2B—H2BB	109.3
C1A—C2A—H2AB	109.4	C1B—C2B—H2BB	109.3
H2AA—C2A—H2AB	108.0	H2BA—C2B—H2BB	108.0
O1A—C3A—C4A	111.35 (16)	O1B—C3B—C4B	111.32 (16)
O1A—C3A—H3AA	109.4	O1B—C3B—H3BA	109.4
C4A—C3A—H3AA	109.4	C4B—C3B—H3BA	109.4
O1A—C3A—H3AB	109.4	O1B—C3B—H3BB	109.4
C4A—C3A—H3AB	109.4	C4B—C3B—H3BB	109.4
H3AA—C3A—H3AB	108.0	H3BA—C3B—H3BB	108.0
N1A—C4A—C3A	109.15 (15)	N1B—C4B—C3B	108.88 (17)
N1A—C4A—H4AA	109.8	N1B—C4B—H4BA	109.9
C3A—C4A—H4AA	109.8	C3B—C4B—H4BA	109.9
N1A—C4A—H4AB	109.8	N1B—C4B—H4BB	109.9
C3A—C4A—H4AB	109.8	C3B—C4B—H4BB	109.9
H4AA—C4A—H4AB	108.3	H4BA—C4B—H4BB	108.3
N1A—C5A—N2A	116.59 (15)	N1B—C5B—N2B	114.58 (15)
N1A—C5A—H5AA	108.1	N1B—C5B—H5BA	108.6
N2A—C5A—H5AA	108.1	N2B—C5B—H5BA	108.6
N1A—C5A—H5AB	108.1	N1B—C5B—H5BB	108.6
N2A—C5A—H5AB	108.1	N2B—C5B—H5BB	108.6
H5AA—C5A—H5AB	107.3	H5BA—C5B—H5BB	107.6
N2A—C6A—N4A	102.74 (14)	N2B—C6B—N4B	102.29 (15)
N2A—C6A—S1A	126.88 (14)	N2B—C6B—S1B	128.04 (14)
N4A—C6A—S1A	130.37 (13)	N4B—C6B—S1B	129.42 (13)
N3A—C7A—N4A	110.73 (15)	N3B—C7B—N4B	110.39 (15)
N3A—C7A—C15A	125.90 (15)	N3B—C7B—C15B	126.41 (15)
N4A—C7A—C15A	123.37 (16)	N4B—C7B—C15B	123.18 (14)
N5A—C8A—C9A	119.57 (16)	N5B—C8B—C9B	119.14 (16)
N5A—C8A—H8AA	120.2	N5B—C8B—H8BA	120.4
C9A—C8A—H8AA	120.2	C9B—C8B—H8BA	120.4
C10A—C9A—C14A	119.39 (17)	C14B—C9B—C10B	120.12 (16)
C10A—C9A—C8A	118.07 (15)	C14B—C9B—C8B	121.56 (16)
C14A—C9A—C8A	122.52 (15)	C10B—C9B—C8B	118.32 (16)
C11A—C10A—C9A	120.99 (16)	C11B—C10B—C9B	120.24 (17)
C11A—C10A—H10A	119.5	C11B—C10B—H10B	119.9
C9A—C10A—H10A	119.5	C9B—C10B—H10B	119.9
C10A—C11A—C12A	117.79 (16)	C12B—C11B—C10B	118.00 (17)
C10A—C11A—H11A	121.1	C12B—C11B—H11B	121.0
C12A—C11A—H11A	121.1	C10B—C11B—H11B	121.0
C11A—C12A—C13A	123.17 (17)	C11B—C12B—C13B	123.11 (16)
C11A—C12A—N6A	118.50 (15)	C11B—C12B—N6B	118.55 (17)
C13A—C12A—N6A	118.33 (16)	C13B—C12B—N6B	118.34 (17)
C14A—C13A—C12A	118.34 (16)	C14B—C13B—C12B	118.41 (17)
C14A—C13A—H13A	120.8	C14B—C13B—H13B	120.8
C12A—C13A—H13A	120.8	C12B—C13B—H13B	120.8
C13A—C14A—C9A	120.31 (16)	C13B—C14B—C9B	120.03 (16)
C13A—C14A—H14A	119.8	C13B—C14B—H14B	120.0
C9A—C14A—H14A	119.8	C9B—C14B—H14B	120.0
C7A—C15A—C16A	111.00 (14)	C7B—C15B—C16B	110.80 (14)
C7A—C15A—C26A	110.41 (16)	C7B—C15B—C26B	110.48 (14)
C16A—C15A—C26A	111.16 (14)	C16B—C15B—C26B	111.55 (15)
C7A—C15A—H15A	108.0	C7B—C15B—H15B	108.0
C16A—C15A—H15A	108.0	C16B—C15B—H15B	108.0
C26A—C15A—H15A	108.0	C26B—C15B—H15B	108.0
C17A—C16A—C21A	118.34 (18)	C21B—C16B—C17B	118.29 (16)
C17A—C16A—C15A	120.76 (16)	C21B—C16B—C15B	120.76 (15)
C21A—C16A—C15A	120.87 (16)	C17B—C16B—C15B	120.92 (15)
C18A—C17A—C16A	120.63 (16)	C18B—C17B—C16B	120.54 (16)
C18A—C17A—H17A	119.7	C18B—C17B—H17B	119.7
C16A—C17A—H17A	119.7	C16B—C17B—H17B	119.7
C17A—C18A—C19A	121.48 (18)	C17B—C18B—C19B	121.29 (16)
C17A—C18A—H18A	119.3	C17B—C18B—H18B	119.4
C19A—C18A—H18A	119.3	C19B—C18B—H18B	119.4
C20A—C19A—C18A	117.81 (18)	C20B—C19B—C18B	117.76 (16)
C20A—C19A—C22A	121.42 (17)	C20B—C19B—C22B	121.53 (16)
C18A—C19A—C22A	120.74 (18)	C18B—C19B—C22B	120.70 (16)
C21A—C20A—C19A	121.09 (16)	C21B—C20B—C19B	121.32 (16)
C21A—C20A—H20A	119.5	C21B—C20B—H20B	119.3
C19A—C20A—H20A	119.5	C19B—C20B—H20B	119.3
C20A—C21A—C16A	120.65 (17)	C20B—C21B—C16B	120.78 (16)
C20A—C21A—H21A	119.7	C20B—C21B—H21B	119.6
C16A—C21A—H21A	119.7	C16B—C21B—H21B	119.6
C19A—C22A—C23A	112.74 (17)	C19B—C22B—C23B	114.17 (14)
C19A—C22A—H22A	109.0	C19B—C22B—H22C	108.7
C23A—C22A—H22A	109.0	C23B—C22B—H22C	108.7
C19A—C22A—H22B	109.0	C19B—C22B—H22D	108.7
C23A—C22A—H22B	109.0	C23B—C22B—H22D	108.7
H22A—C22A—H22B	107.8	H22C—C22B—H22D	107.6
C25A—C23A—C24A	111.04 (19)	C24B—C23B—C25B	111.29 (15)
C25A—C23A—C22A	111.87 (18)	C24B—C23B—C22B	109.62 (15)
C24A—C23A—C22A	110.0 (2)	C25B—C23B—C22B	110.98 (15)
C25A—C23A—H23A	107.9	C24B—C23B—H23B	108.3
C24A—C23A—H23A	107.9	C25B—C23B—H23B	108.3
C22A—C23A—H23A	107.9	C22B—C23B—H23B	108.3
C23A—C24A—H24A	109.5	C23B—C24B—H24D	109.5
C23A—C24A—H24B	109.5	C23B—C24B—H24E	109.5
H24A—C24A—H24B	109.5	H24D—C24B—H24E	109.5
C23A—C24A—H24C	109.5	C23B—C24B—H24F	109.5
H24A—C24A—H24C	109.5	H24D—C24B—H24F	109.5
H24B—C24A—H24C	109.5	H24E—C24B—H24F	109.5
C23A—C25A—H25A	109.5	C23B—C25B—H25D	109.5
C23A—C25A—H25B	109.5	C23B—C25B—H25E	109.5
H25A—C25A—H25B	109.5	H25D—C25B—H25E	109.5
C23A—C25A—H25C	109.5	C23B—C25B—H25F	109.5
H25A—C25A—H25C	109.5	H25D—C25B—H25F	109.5
H25B—C25A—H25C	109.5	H25E—C25B—H25F	109.5
C15A—C26A—H26A	109.5	C15B—C26B—H26D	109.5
C15A—C26A—H26B	109.5	C15B—C26B—H26E	109.5
H26A—C26A—H26B	109.5	H26D—C26B—H26E	109.5
C15A—C26A—H26C	109.5	C15B—C26B—H26F	109.5
H26A—C26A—H26C	109.5	H26D—C26B—H26F	109.5
H26B—C26A—H26C	109.5	H26E—C26B—H26F	109.5
			
C6A—N2A—N3A—C7A	1.26 (19)	C6B—N2B—N3B—C7B	0.14 (19)
C5A—N2A—N3A—C7A	170.29 (15)	C5B—N2B—N3B—C7B	−169.00 (15)
C7A—N4A—N5A—C8A	−178.80 (16)	C7B—N4B—N5B—C8B	−154.02 (16)
C6A—N4A—N5A—C8A	4.7 (3)	C6B—N4B—N5B—C8B	27.8 (2)
C5A—N1A—C1A—C2A	−173.33 (17)	C5B—N1B—C1B—C2B	176.37 (17)
C4A—N1A—C1A—C2A	56.5 (2)	C4B—N1B—C1B—C2B	−55.0 (2)
C3A—O1A—C2A—C1A	60.5 (2)	C3B—O1B—C2B—C1B	−58.5 (2)
N1A—C1A—C2A—O1A	−58.7 (2)	N1B—C1B—C2B—O1B	56.2 (2)
C2A—O1A—C3A—C4A	−60.2 (2)	C2B—O1B—C3B—C4B	61.0 (2)
C5A—N1A—C4A—C3A	173.79 (16)	C5B—N1B—C4B—C3B	−175.09 (15)
C1A—N1A—C4A—C3A	−56.4 (2)	C1B—N1B—C4B—C3B	56.5 (2)
O1A—C3A—C4A—N1A	58.0 (2)	O1B—C3B—C4B—N1B	−60.3 (2)
C1A—N1A—C5A—N2A	−58.7 (2)	C1B—N1B—C5B—N2B	52.5 (2)
C4A—N1A—C5A—N2A	69.8 (2)	C4B—N1B—C5B—N2B	−74.16 (19)
C6A—N2A—C5A—N1A	−98.3 (2)	C6B—N2B—C5B—N1B	−90.8 (2)
N3A—N2A—C5A—N1A	94.32 (18)	N3B—N2B—C5B—N1B	77.1 (2)
N3A—N2A—C6A—N4A	−1.32 (19)	N3B—N2B—C6B—N4B	−2.46 (18)
C5A—N2A—C6A—N4A	−169.22 (16)	C5B—N2B—C6B—N4B	166.25 (15)
N3A—N2A—C6A—S1A	177.59 (13)	N3B—N2B—C6B—S1B	172.11 (12)
C5A—N2A—C6A—S1A	9.7 (3)	C5B—N2B—C6B—S1B	−19.2 (2)
N5A—N4A—C6A—N2A	177.62 (17)	C7B—N4B—C6B—N2B	3.77 (17)
C7A—N4A—C6A—N2A	0.86 (18)	N5B—N4B—C6B—N2B	−177.93 (15)
N5A—N4A—C6A—S1A	−1.2 (3)	C7B—N4B—C6B—S1B	−170.69 (13)
C7A—N4A—C6A—S1A	−177.99 (14)	N5B—N4B—C6B—S1B	7.6 (3)
N2A—N3A—C7A—N4A	−0.63 (19)	N2B—N3B—C7B—N4B	2.35 (18)
N2A—N3A—C7A—C15A	179.58 (16)	N2B—N3B—C7B—C15B	−178.88 (15)
N5A—N4A—C7A—N3A	−177.44 (14)	C6B—N4B—C7B—N3B	−4.03 (19)
C6A—N4A—C7A—N3A	−0.1 (2)	N5B—N4B—C7B—N3B	177.45 (14)
N5A—N4A—C7A—C15A	2.3 (2)	C6B—N4B—C7B—C15B	177.16 (15)
C6A—N4A—C7A—C15A	179.64 (16)	N5B—N4B—C7B—C15B	−1.4 (2)
N4A—N5A—C8A—C9A	179.47 (15)	N4B—N5B—C8B—C9B	179.34 (14)
N5A—C8A—C9A—C10A	−179.63 (17)	N5B—C8B—C9B—C14B	−8.0 (3)
N5A—C8A—C9A—C14A	−1.0 (3)	N5B—C8B—C9B—C10B	172.27 (17)
C14A—C9A—C10A—C11A	−0.4 (3)	C14B—C9B—C10B—C11B	0.6 (3)
C8A—C9A—C10A—C11A	178.28 (17)	C8B—C9B—C10B—C11B	−179.69 (17)
C9A—C10A—C11A—C12A	1.0 (3)	C9B—C10B—C11B—C12B	1.8 (3)
C10A—C11A—C12A—C13A	−0.7 (3)	C10B—C11B—C12B—C13B	−2.1 (3)
C10A—C11A—C12A—N6A	179.12 (16)	C10B—C11B—C12B—N6B	178.35 (18)
O2A—N6A—C12A—C11A	21.0 (2)	O2B—N6B—C12B—C11B	27.0 (3)
O3A—N6A—C12A—C11A	−158.05 (18)	O3B—N6B—C12B—C11B	−152.71 (19)
O2A—N6A—C12A—C13A	−159.17 (17)	O2B—N6B—C12B—C13B	−152.5 (2)
O3A—N6A—C12A—C13A	21.8 (3)	O3B—N6B—C12B—C13B	27.8 (3)
C11A—C12A—C13A—C14A	−0.2 (3)	C11B—C12B—C13B—C14B	0.0 (3)
N6A—C12A—C13A—C14A	179.95 (16)	N6B—C12B—C13B—C14B	179.46 (17)
C12A—C13A—C14A—C9A	0.8 (3)	C12B—C13B—C14B—C9B	2.5 (3)
C10A—C9A—C14A—C13A	−0.5 (3)	C10B—C9B—C14B—C13B	−2.8 (3)
C8A—C9A—C14A—C13A	−179.18 (17)	C8B—C9B—C14B—C13B	177.46 (16)
N3A—C7A—C15A—C16A	−99.4 (2)	N3B—C7B—C15B—C16B	−113.39 (18)
N4A—C7A—C15A—C16A	80.9 (2)	N4B—C7B—C15B—C16B	65.2 (2)
N3A—C7A—C15A—C26A	24.4 (2)	N3B—C7B—C15B—C26B	10.8 (2)
N4A—C7A—C15A—C26A	−155.41 (16)	N4B—C7B—C15B—C26B	−170.62 (15)
C7A—C15A—C16A—C17A	−122.66 (17)	C7B—C15B—C16B—C21B	47.1 (2)
C26A—C15A—C16A—C17A	114.03 (18)	C26B—C15B—C16B—C21B	−76.4 (2)
C7A—C15A—C16A—C21A	59.1 (2)	C7B—C15B—C16B—C17B	−134.77 (17)
C26A—C15A—C16A—C21A	−64.3 (2)	C26B—C15B—C16B—C17B	101.69 (19)
C21A—C16A—C17A—C18A	0.6 (2)	C21B—C16B—C17B—C18B	−0.5 (3)
C15A—C16A—C17A—C18A	−177.77 (15)	C15B—C16B—C17B—C18B	−178.69 (17)
C16A—C17A—C18A—C19A	0.3 (3)	C16B—C17B—C18B—C19B	−0.7 (3)
C17A—C18A—C19A—C20A	−1.0 (3)	C17B—C18B—C19B—C20B	1.3 (3)
C17A—C18A—C19A—C22A	177.05 (16)	C17B—C18B—C19B—C22B	−179.32 (17)
C18A—C19A—C20A—C21A	0.9 (3)	C18B—C19B—C20B—C21B	−0.7 (3)
C22A—C19A—C20A—C21A	−177.15 (16)	C22B—C19B—C20B—C21B	179.96 (17)
C19A—C20A—C21A—C16A	−0.1 (3)	C19B—C20B—C21B—C16B	−0.5 (3)
C17A—C16A—C21A—C20A	−0.7 (2)	C17B—C16B—C21B—C20B	1.1 (3)
C15A—C16A—C21A—C20A	177.66 (15)	C15B—C16B—C21B—C20B	179.32 (17)
C20A—C19A—C22A—C23A	102.2 (2)	C20B—C19B—C22B—C23B	105.08 (19)
C18A—C19A—C22A—C23A	−75.7 (2)	C18B—C19B—C22B—C23B	−74.2 (2)
C19A—C22A—C23A—C25A	−60.2 (2)	C19B—C22B—C23B—C24B	−179.82 (14)
C19A—C22A—C23A—C24A	175.88 (19)	C19B—C22B—C23B—C25B	−56.47 (19)

### Hydrogen-bond geometry (Å, °)

**Table:**

*D*—H···*A*	*D*—H	H···*A*	*D*···*A*	*D*—H···*A*
C4A—H4AB···O3A^i^	0.97	2.53	3.450 (3)	157
C5A—H5AB···O3B^ii^	0.97	2.56	3.221 (3)	126
C8A—H8AA···S1A	0.93	2.43	3.186 (2)	138
C13A—H13A···O3A^iii^	0.93	2.43	3.064 (3)	125
C13B—H13B···N1A^iv^	0.93	2.55	3.332 (2)	141
C15A—H15A···O1A^v^	0.98	2.27	3.212 (2)	161
C5B—H5BA···S1B	0.97	2.83	3.252 (2)	107
C8B—H8BA···S1B	0.93	2.55	3.1823 (19)	125
C22B—H22D···S1A^iv^	0.97	2.86	3.801 (2)	163
N6A—O2A···Cg1^i^	1.225 (2)	3.0012 (17)	3.6928 (19)	115.19 (12)
C11A—H11A···Cg2^i^	0.93	2.52	3.405 (2)	158

Symmetry codes: (i) −*x*+1, −*y*+1, −*z*+2; (ii) *x*+1, *y*, *z*+1;  (iii) −*x*, −*y*+1, −*z*+2; (iv) *x*−1, *y*, *z*−1; (v) *x*−1, *y*, *z*.
